# Effects of 12-Week Multiple Micronutrient Supplementation on Immune Function, Body Weight, and Metabolic Parameters in Middle-Aged Women: A Retrospective Study

**DOI:** 10.7759/cureus.92449

**Published:** 2025-09-16

**Authors:** Yu Sun

**Affiliations:** 1 Functional Medicine and Integrative Medicine, NingDa Longevity Clinic, Beijing, CHN

**Keywords:** body composition, immune function, micronutrient, vitamin, weight loss

## Abstract

Objective: This retrospective study aimed to evaluate the effects of a 12-week combined supplementation of vitamin D3, vitamin K2, vitamin B6, vitamin B12, and magnesium on immune function, body composition, and metabolic parameters in women aged 45 years and older with suboptimal micronutrient intake, using real-world electronic health record (EHR) data.

Methods: De-identified EHR data from 52 Chinese women (mean age: 49.2 ± 5.8 years) who received a daily standardized supplement (vitamin D3: 5000 IU, vitamin K2: 100 μg, vitamin B6: 2.5 mg, vitamin B12: 1000 μg, magnesium: 75 mg) for 12 consecutive weeks were analyzed. Outcomes included immune markers (immunoglobulins, high-sensitivity CRP, WBC count with differential), anthropometric measures (body weight, BMI, body fat percentage, waist circumference), and metabolic parameters (homocysteine, fasting glucose, lipid profile) at baseline and 12 weeks post-supplementation.

Results: After 12 weeks, significant improvements were observed in immune function: serum IgG increased from 9.8 ± 1.7 g/L to 11.3 ± 1.5 g/L (p<0.01), IgA rose from 2.0 ± 0.4 g/L to 2.5 ± 0.3 g/L (p<0.01), and high-sensitivity CRP decreased from 3.5 ± 1.2 mg/L to 2.2 ± 0.9 mg/L (p<0.001). Anthropometric changes included modest but significant reductions in body weight (−1.4 ± 0.7 kg, p<0.01), body fat percentage (−1.2 ± 0.5%, p<0.01), and waist circumference (−1.5 ± 0.6 cm, p<0.01). Metabolic health showed marked improvements: fasting glucose decreased from 93.5 ± 6.4 mg/dL to 89.8 ± 5.8 mg/dL (p<0.05), total cholesterol declined from 201 ± 23 mg/dL to 188 ± 20 mg/dL (p<0.05), and homocysteine dropped from 11.2 ± 2.3 μmol/L to 8.5 ± 1.8 μmol/L (p<0.01). No adverse events were reported.

Conclusions: Twelve-week supplementation with vitamin D3, K2, B6, B12, and magnesium improves immune function, reduces adiposity while preserving lean mass, and ameliorates metabolic dysregulation in middle-aged women. This combined regimen may serve as a promising strategy to address midlife-related health risks.

## Introduction

Micronutrients, including vitamins and minerals, play critical roles in immune function, energy metabolism, and maintaining healthy body composition [1]. Despite advances in food availability, suboptimal intake of key micronutrients remains prevalent globally, particularly among women aged 45 years and older who face unique physiological challenges related to menopausal transition [2]. Suboptimal intake of micronutrients in midlife and older women is linked to a cascade of health risks, including elevated cardiovascular disease risk, systemic inflammation, age-related weight gain, and impaired immune responses to infections [3].

Multiple micronutrient supplements are considered to have the potential to address these issues. However, their clinical efficacy remains debated, with conflicting results from randomized controlled trials [4]. Retrospective studies using electronic health record (EHR) data offer valuable insights into the actual effectiveness of multiple micronutrient supplementations in real-world settings. This retrospective study aimed to investigate the effects of a 12-week combined supplementation regimen targeting five critical micronutrients (vitamin D, vitamin K, vitamin B6, vitamin B12, and magnesium) in middle-aged women by analyzing EHR data from a clinic, with the purpose of clarifying whether the supplementation could improve immune function, body weight, and metabolic parameters in a real-world setting.

## Materials and methods

Study design and participants

This retrospective study used de-identified EHR data from NingDa Longevity Clinic, a functional medicine and integrative medicine clinic in Beijing, China, collected between October 2024 and August 2025. The study protocol was approved by the Institutional Review Board of Ningda Clinic.

Eligible participants were women aged 45 years and older who met the following criteria: i) Received a standardized daily supplement containing vitamin D3 (5000IU), vitamin K2 (100μg), vitamin B6 (2.5mg), vitamin B12 (1000μg), and magnesium (75mg) for 12 consecutive weeks; ii) Had baseline (pre-supplementation) and 12-week post-supplementation measurements of immune markers, anthropometrics, and metabolic parameters in EHRs; iii) No chronic diseases (e.g., type 2 diabetes, autoimmune disorders, chronic kidney disease) or use of medications that interact with the supplement (e.g., warfarin for vitamin K, metformin for vitamin B12) during the supplementation period; iv) No acute infections (e.g., influenza, urinary tract infection) four weeks before baseline or post-supplementation assessments. Exclusion criteria included: i) Missing baseline or post-supplementation data; ii) Non-adherence to supplementation; iii) Concurrent use of other micronutrient supplements.

Data extraction

Data were extracted from EHRs at baseline and after 12 weeks of supplementation. Outcome measures included demographic characteristics (age, ethnicity, education level, and baseline dietary intake), immune markers (serum immunoglobulins, high-sensitivity C-reactive protein (CRP), total white blood cell (WBC) count with differential), anthropometrics (body weight, height, body fat percentage, and waist circumference), and metabolic parameters (fasting glucose, and lipid profile).

Statistical analysis

Normality of distribution was assessed using the Shapiro-Wilk test. Baseline and post-supplementation comparisons were made using paired t-tests for normally distributed data and Wilcoxon signed-rank tests for non-normal distributions. Results are presented as mean ± standard deviation. Statistical significance was set at p<0.05. Data were analyzed using IBM SPSS Statistics for Windows, Version 26 (Released 2019; IBM Corp., Armonk, New York, United States).

## Results

A total of 52 Chinese women were included in the final analysis (mean age: 49.2 ± 5.8 years; range: 45-63 years). The majority of the participants (69.2%, n=36) were post-menopausal. Regarding the education level, 41 participants (78.8%) had a college or graduate degree. The mean baseline BMI of the participants was 25.3 ± 3.1 kg/m^2^. Most participants (80.7%, n=42) had suboptimal intake of two or more target micronutrients at baseline. No adverse events such as gastrointestinal discomfort or bruising were documented during the supplementation.

In terms of immune markers, notable improvements were observed after the 12-week intervention. Serum levels of two key immunoglobulins involved in humoral immunity, IgG and IgA, increased significantly, while the inflammatory marker high-sensitivity CRP decreased substantially, indicating reduced systemic inflammation. Total WBC count showed a modest, non-significant upward trend, but the percentage of lymphocytes rose significantly. In contrast, serum IgM levels and neutrophil percentage remained stable with no significant changes (Table 1).

**Table 1:** Changes in Immune Markers, Anthropometrics, and Metabolic Parameters (n=52) WBC: white blood cell; CRP: C-reactive protein; LDL: low-density lipoprotein; HDL: high-density lipoprotein; Δ: change from the baseline to 12 weeks.

Parameter	Baseline	12 Weeks Post-supplementation	Δ (Mean ± SD)	p-value	Cohen’s d
Immune Markers					
IgG (g/L)	9.8 ± 1.7	11.3 ± 1.5	+1.5 ± 1.0	<0.01	0.92
IgA (g/L)	2.0 ± 0.4	2.5 ± 0.3	+0.5 ± 0.3	<0.01	0.98
IgM (g/L)	0.8 ± 0.2	0.9 ± 0.2	+0.1 ± 0.2	0.14	0.39
CRP (mg/L)	3.5 ± 1.2	2.2 ± 0.9	-1.3 ± 0.8	<0.01	1.28
Total WBC (×10⁹/L)	5.6 ± 1.1	6.0 ± 1.0	+0.4 ± 0.7	0.07	0.33
Lymphocyte (%)	31.5 ± 5.2	34.5 ± 4.7	+3.0 ± 3.4	<0.05	0.61
Anthropometrics					
Body weight (kg)	74.6 ± 10.5	73.2 ± 10.0	-1.4 ± 0.7	<0.01	0.37
BMI (kg/m²)	25.3 ± 3.1	24.8 ± 3.0	-0.5 ± 0.2	<0.01	0.38
Body fat (%)	26.4 ± 5.3	25.2 ± 5.0	-1.2 ± 0.5	<0.01	0.45
Waist circumference (cm)	90.2 ± 8.5	88.7 ± 8.0	-1.5 ± 0.6	<0.01	0.46
Metabolic Parameters					
Homocysteine (μmol/L)	11.2 ± 2.3	8.5 ± 1.8	-2.7 ± 1.9	<0.01	1.21
Fasting glucose (mg/dL)	93.5 ± 6.4	89.8 ± 5.8	-3.7 ± 3.6	<0.05	0.55
Total cholesterol (mg/dL)	201 ± 23	188 ± 20	-13 ± 16	<0.05	0.51
LDL cholesterol (mg/dL)	125 ± 19	118 ± 17	-7 ± 12	<0.05	0.42
HDL cholesterol (mg/dL)	53 ± 8	54 ± 7	+1 ± 6	0.39	0.12
Triglycerides (mg/dL)	132 ± 47	125 ± 42	-7 ± 33	0.29	0.16

For anthropometric measures, the participants experienced significant positive changes in body composition and size. Body weight and BMI both decreased significantly, accompanied by a notable reduction in body fat percentage, suggesting a loss of fat mass rather than just overall weight. Waist circumference also declined significantly. Importantly, lean mass was preserved throughout the intervention, with no significant changes observed, which is clinically relevant for maintaining muscle health (Table 1).

Regarding metabolic parameters, the 12-week supplementation led to significant improvements in key indicators of metabolic health. Serum homocysteine, a marker closely linked to B-vitamin status and cardiovascular risk, decreased markedly. Fasting glucose levels and total cholesterol also dropped significantly. Additionally, low-density lipoprotein (LDL) cholesterol showed a significant reduction, while high-density lipoprotein (HDL) cholesterol and triglyceride levels remained unchanged with no significant fluctuations (Table 1).

## Discussion

This retrospective study using real-world EHR data demonstrates that a 12-week combined supplementation regimen with vitamin D3, vitamin K2, vitamin B6, vitamin B12, and magnesium produces significant improvements in immune function, body composition, and metabolic parameters in women aged 45 years and older.

The increases in IgG and IgA align with the combined effects of vitamin D3 and magnesium on humoral immunity. Vitamin D3 activates vitamin D receptors on B cells, promoting their differentiation into antibody-secreting plasma cells, while magnesium supports B-cell proliferation and prevents immune cell apoptosis induced by oxidative stress [5,6]. For middle-aged women, declining estrogen levels weaken antibody production, making these micronutrient-driven improvements particularly clinically relevant for infection prevention. The reduction in CRP highlights the anti-inflammatory effects mediated by multiple micronutrient supplementation. The modest rise in lymphocyte percentage further supports enhanced adaptive immunity, a critical defense against age-related immune senescence [7]. IgM levels remained unchanged, likely due to the shorter intervention duration.

The weight loss and reduction in body fat percentage are clinically meaningful for middle-aged women, as even small adiposity reductions lower the risk of menopause-related metabolic disorders [8]. These changes are driven by magnesium and B vitamins. Magnesium improves insulin sensitivity, limiting fat storage in visceral tissues by enhancing glucose uptake into muscle cells [9]. Vitamins B6 and B12 act as coenzymes in fatty acid oxidation, increasing energy utilization and reducing fat accumulation. The preservation of lean mass is attributed to magnesium, which supports muscle protein synthesis by regulating calcium-dependent muscle contraction, and vitamin D3, which maintains muscle fiber size [10]. For middle-aged women, preserving lean mass is essential for preventing sarcopenia and maintaining mobility.

The reduction in homocysteine is mediated by vitamins B6 and B12. Vitamin B6 acts as a coenzyme in homocysteine transsulfuration to cysteine, while vitamin B12 supports homocysteine remethylation to methionine [11]. Magnesium further enhances this pathway by activating B6-dependent enzymes, explaining the more pronounced homocysteine reduction compared to single vitamin B supplementation [12]. The reductions in fasting glucose and LDL cholesterol address two major CVD risk factors. Magnesium drives glucose lowering by regulating insulin receptor signaling in skeletal muscle, while vitamin D3 improves pancreatic β-cell function. For lipids, vitamin K2 reduces LDL cholesterol oxidation by inhibiting vascular calcification, and magnesium enhances HDL-mediated cholesterol clearance [13].

This study has several limitations inherent to its retrospective design. The lack of a control group prevents definitive attribution of the observed effects to the intervention alone. The relatively small sample size and short intervention period limit generalizability. Additionally, the single-arm design prevents examination of potential placebo effects. Future research should address these limitations through randomized controlled trials with larger sample sizes and longer follow-up periods.

## Conclusions

This retrospective study demonstrates that 12-week supplementation with vitamin D3, vitamin K2, B6, B12, and magnesium improves immune function, body composition, and metabolic parameters in women aged 45 years and older with suboptimal intake of these nutrients. The synergistic effects of combined nutrient supplementation may offer advantages over single-nutrient approaches for addressing the complex physiological changes that occur during midlife and beyond. While further research is needed to confirm these findings in randomized controlled trials, the results suggest that multiple micronutrient supplementation represents a promising strategy for supporting immune function, metabolic health, and healthy body composition in middle-aged women.
